# Long Astral Microtubules and RACK-1 Stabilize Polarity Domains during Maintenance Phase in *Caenorhabditis elegans* Embryos

**DOI:** 10.1371/journal.pone.0019020

**Published:** 2011-04-20

**Authors:** Erkang Ai, Daniel S. Poole, Ahna R. Skop

**Affiliations:** Laboratory of Genetics, University of Wisconsin-Madison, Madison, Wisconsin, United States of America; University of Colorado, Boulder, United States of America

## Abstract

Cell polarity is a very well conserved process important for cell differentiation, cell migration, and embryonic development. After the establishment of distinct cortical domains, polarity cues have to be stabilized and maintained within a fluid and dynamic membrane to achieve proper cell asymmetry. Microtubules have long been thought to deliver the signals required to polarize a cell. While previous studies suggest that microtubules play a key role in the establishment of polarity, the requirement of microtubules during maintenance phase remains unclear. In this study, we show that depletion of *Caenorhabditis elegans* RACK-1, which leads to short astral microtubules during prometaphase, specifically affects maintenance of cortical PAR domains and Dynamin localization. We then investigated the consequence of knocking down other factors that also abolish astral microtubule elongation during polarity maintenance phase. We found a correlation between short astral microtubules and the instability of PAR-6 and PAR-2 domains during maintenance phase. Our data support a necessary role for astral microtubules in the maintenance phase of cell polarity.

## Introduction

Polarity plays a key role in cell differentiation, cell fate determination, cell motility, and development. The *Caenorhabditis elegans* one-cell embryos become polarized shortly after fertilization. Antagonistic PAR proteins generate two distinct cortical domains [1]. PAR-6, PAR-3, and PKC-3 constitute the anterior PAR complex, while PAR-2 localizes to the posterior cortex by the exclusion of PAR-3 [2]. These PAR protein localizations form two non-overlapping domains [3]. Polarization along the anterior-posterior (AP) axis results in differences in cortical actomyosin organization, membrane tension, and astral microtubule pulling forces [4], [5], [6]. These events eventually lead to the displacement of the mitotic spindle, resulting in an asymmetry both in daughter cell size and cell fate [7], [8].

The polarization in one-cell *C. elegans* embryos can be divided into two phases, establishment and maintenance [9], [10]. The initial cue to induce polarity is an unknown centrosome-dependent signal [11], [12], [13], [14]. This signal destabilizes the acto-myosin network in the posterior cortex where the sperm centrosome resides [15], resulting in a cortical flow that transports actin as well as anterior PAR complex towards the anterior [16], [17], [18]. The establishment of polarity is completed before the maternal pronucleus starts to migrate to the posterior, and the hallmark of completed establishment phase is pseudocleavage furrow formation [10], [15], [19]. The localization of PAR-6/PAR-3/PKC-3 and PAR-2 is stabilized in defined regions of the cortex throughout mitosis. The maintenance of polarity requires a balance of membrane fusion and endocytosis [16], [20].

Microtubules are ideal for transporting the potential symmetry-breaking signals generated by the sperm centrosome to the cortex and have been suggested to be involved in polarity establishment in multiple organisms [21]. In *C. elegans* embryos, however, the requirement of microtubules in establishing polarity has been controversial. Although earlier studies using mutants defective in centrosome maturation (*spd-5, spd-2)* suggested a role for microtubules in inducing posterior polarity [13], [14], [22], other studies using nocodazole and *tbb-2* RNA interference (RNAi) knockdowns suggested that the centrosome may induce polarity independently of microtubules [11], [23]. Later studies revisited the anterior PAR-2 localization in the *spd-5* mutant as well as the consequences of *tbb-2* RNAi knockdown and concluded that microtubules are involved in the symmetry breaking event [24]. Despite these findings, it is unclear whether microtubules also play a similar role during the maintenance phase of polarity.

In this study, we explored the relationship between microtubules, particularly astral microtubules, and the maintenance of polarity in *C. elegans* embryos. We identified a strong correlation between astral microtubule length and the stabilization of cortical polarity domains. Our results support a role for astral microtubules in the maintenance phase of polarity.

## Materials and Methods

### Worm strains

The following strains were used: JJ1579 (PAR-6-GFP) [25], TH129 (GFP-PAR-2) [26], TH120 (GFP-PAR-2; mCherry-PAR-6) [26], WH204 (GFP-TBB-2) [27], MAD27 (GFP-TBB-2; GFP-PAR-2; mCherry-PAR-6) (this study). Strain MAD27 was obtained by crossing strain TH120 with WH204. Worms were maintained and cultured at 25°C as described by Brenner [28].

### RNA interference

RNA interference (RNAi) was performed by the feeding method (Timmons *et al.*, 2001). L4-stage hermaphrodites were fed bacteria expressing double-stranded RNA. *rack-1, tbb-2, zyg-9*, and *zen-4* RNAi feeding bacteria were obtained from the Ahringer RNAi library [29] and sequence verified. *rab-11* and *dnc-2* RNAi feeding bacteria were obtained as previously described [30]. *rack-1(RNAi)* and *dnc-2(RNAi)* experiments were performed for 40–48 hours at 20°C, or 30–36 hours at 25°C. Other RNAi experiments were performed for 20–25 hours at 20°C. Complete depletion of RAB-11 results in decreased brood size and sterility due to defects in embryogenesis and germline membrane organization. Therefore, a 1∶1 ratio of bacterial cultures containing *rab-11(RNAi)* plasmid and L4440 vector were mixed together to reduce the RNAi effect.

### Live imaging

Embryos were dissected in 10 µl Shelton's Growth Media [31] on a 22 mm×22 mm coverslip. A 2% agarose pad in egg salts (118 mM NaCl, 40 mM KCl, 3.4 mM CaCl_2_, 3.4 mM MgCl_2_, 5 mM HEPES [pH 7.2]) [15] was placed on top of the coverslips and sealed with Vaseline. Time-lapse videos were recorded using a Zeiss 200 M inverted Axioskop microscope equipped with a spinning disk confocal scan head (QLC100, Visitech International). The motorized filter turret and focus, external shutters, and a 12-bit camera (Orca ER; Hamamatsu) were controlled using OpenLab software (Improvision, Inc). Sequential images were acquired every 20 seconds using a 63×, 1.4 NA Plan-Apochromat objective. The exposure times for each strain were as follows: 300 ms (GFP-PAR-2 and DYN-1-GFP cortical movies), 500 ms (PAR-6-GFP cortical movies). For imaging the GFP-TBB-2; GFP-PAR-2; mCherry-PAR-6 strain, the exposure time for GFP-TBB-2 and GFP-PAR-2 was 400 ms, and 1300 ms for mCherry-PAR-6. For cortical time-lapse imaging, we collected a Z-series of 4 frames at 0.5 µm step per time point, and converted each Z-series into a single image by maximum projection. Image processing was done with Adobe PhotoShop and ImageJ software [32].

### Fluorescence intensity analysis

To analyze the effect of *rack-1(RNAi)* on cortical PAR-6-GFP, GFP-PAR-2, and DYN-1-GFP distribution, the cortical projection of the embryo was divided into anterior and posterior sections bordered by the pseudocleavage furrow. Cortical polarities are completely established with the formation of the pseudocleavage furrow and are maintained through the cell cycle. Thus we measured the average fluorescence intensity in the anterior and the posterior regions at the presence of the pseudocleavage furrow for the establishment time point, at seven minutes after the retraction of the pseudocleavage furrow for the maintenance time point, at furrow initiation, and at furrow completion. The average ratio of signal intensity in the anterior half to that in the posterior half was then plotted. For PAR-6-GFP embryos, N = 7 embryos were analyzed. For GFP-PAR-2 embryos, N = 10 embryos were analyzed. For DYN-1-GFP embryos, N = 10 embryos were analyzed.

### Microtubule length and polarity shift analysis

To estimate the lengths of the astral microtubules, images from control embryos and embryos with different RNAi treatments were processed in ImageJ to enhance the contrast for easier visualization of microtubule bundles. For each embryo, the lengths of the five longest astral microtubule bundles at nuclear envelope breakdown (NEBD) were measured in ImageJ. The lengths were then averaged for each embryo and plotted.

To quantify the shift of boundaries between the PAR-2 and PAR-6 domains, mid-focal-plane images of embryos at NEBD stage (the first frame as soon as the breakdown of pronuclear envelopes began) were selected for control and each RNAi treatment. In *tbb-2(RNAi)* and *zyg-9(RNAi)* embryos, the posterior migration of the oocyte pronucleus was delayed, so that the paternal pronucleus underwent NEBD prior to pronuclear meeting and later formed a spindle without the oocyte pronucleus. In these embryos the first frame after the breakdown of the paternal pronuclear envelope was selected for measurement. For each embryo, the boundaries between the PAR-2 and PAR-6 domains were determined by eye. A line was drawn to connect the two boundary points at the sides of the embryo. The angle formed between this line and the dorsal-ventral axis (assumed to be perpendicular to the anterior-posterior axis of the embryo) was measured in ImageJ. Determination of boundaries and measurements of angles were performed on relabeled images in a single-blind analysis.

### Supplementary Data

Videos S1, S2, S3, S4, S5, S6, S7, S8, S9, S10, S11, S12, and S13 are available online. For all videos, anterior is to the left and posterior to the right. Frames were captured 20 seconds apart and play back at 7 frames per second. Cortical Z-series projections used 4 slices with a step size of 0.5 µm.

## Results

### RACK-1 is required for the localization of PAR proteins during maintenance phase but not establishment phase

**Figure 1:**
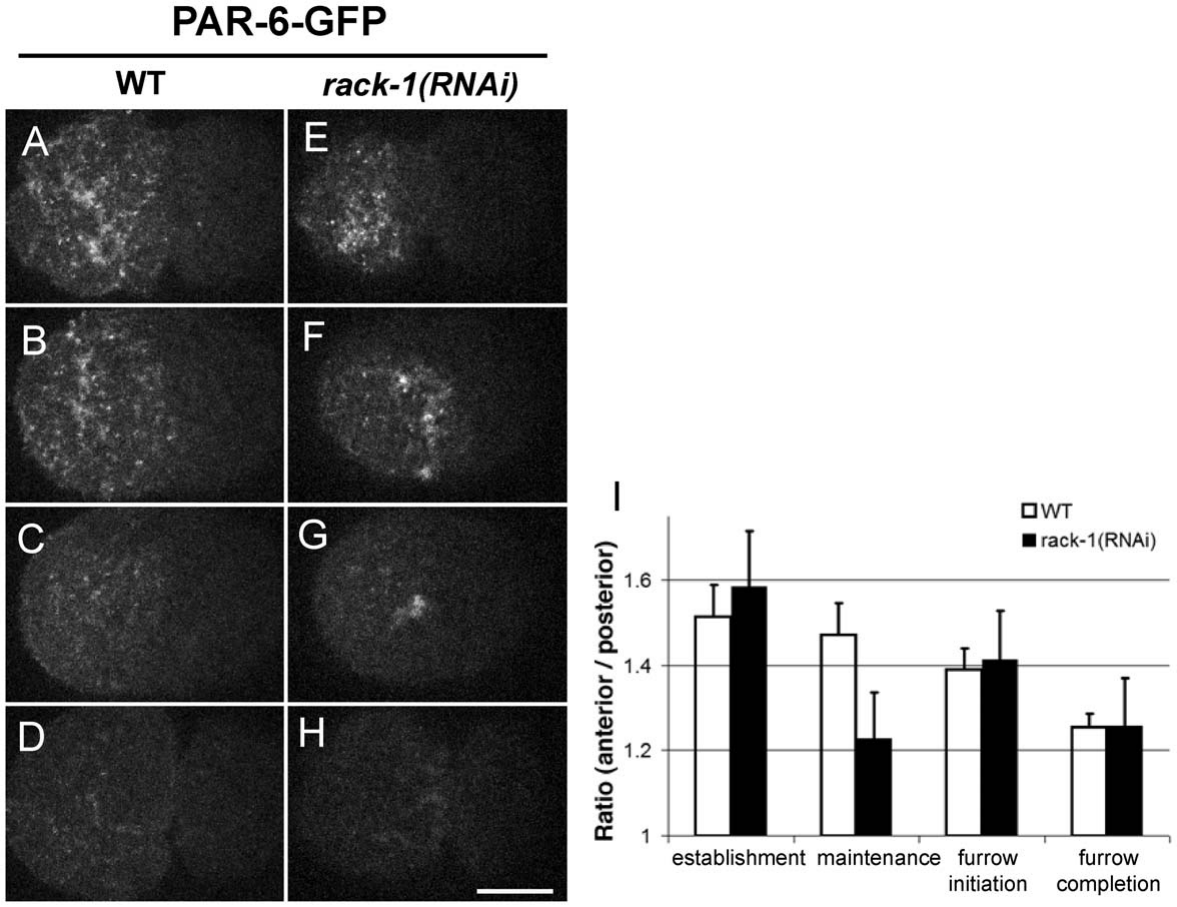
RACK-1 is required for localization of cortical PAR-6-GFP. Selected cortical projections from confocal time-lapse videos show PAR-6-GFP localization in control untreated embryos (A–D; Video S1) and embryos from *rack-1(RNAi)* worms (E–H; Video S2). For each video, four images are selected to represent establishment phase (pseudocleavage, A and E), maintenance phase (about seven minutes after retraction of the pseudocleavage furrow, B and F), cleavage furrow initiation (C and G), and furrow completion (D and H). In all images, anterior is to the left and posterior is to the right. (I) Quantification of the ratios between average PAR-6-GFP intensity in the anterior and posterior halves. Four time points are measured corresponding to the four rows in A–H. White bars represent data from control embryos and black bars represent data from *rack-1(RNAi)* embryos. (Scale bar: 10 µm; error bars: SEM).

In our study, we found that RACK-1, a scaffolding protein that is required for cytokinesis [30], plays a role in maintaining the cortical localization of polarity proteins, specifically PAR proteins, during the asymmetric cell division of one-cell embryos. We recorded time-lapse cortical Z-series images in strains expressing PAR-6-GFP or GFP-PAR-2, which mark the anterior or posterior cortex, respectively [10], [26]. In control untreated PAR-6-GFP embryos, small PAR-6-GFP puncta moved towards the anterior along with the cortical flow (Fig. 1A, polarity establishment phase; Video S1). PAR-6-GFP foci became enriched in the anterior half as the pseudocleavage furrow formed. This anterior enrichment was maintained stable throughout pronuclear meeting, centration, and rotation (Fig. 1B, polarity maintenance phase). The cleavage furrow formed slightly posterior to the middle of the embryo, corresponding to the boundary of the PAR-6-GFP enriched region (Fig. 1C). After completion of cytokinesis, the PAR-6-GFP foci remained enriched in the larger AB daughter cell (Fig. 1D). In *rack-1(RNAi)* embryos, the establishment of PAR-6 foci was not affected, as PAR-6-GFP was normally enriched in the anterior half of the embryo by pseudocleavage formation (Fig. 1E). However, during pronuclear centration and rotation, PAR-6-GFP was not restricted to the anterior cortex but expanded into the posterior half of the cortex (4/7 embryos, Fig. 1F; Video S2). The boundary of PAR-6-GFP was sometimes rotated (2/7 embryos). The expansion or rotation of the PAR-6 domain was corrected during anaphase, just prior to furrow initiation, when PAR-6-GFP localization was indistinguishable from that observed in control embryos. The cleavage furrow was properly placed (Fig. 1G). We also noticed clumps of PAR-6-GFP foci during maintenance phase and furrow formation (Fig. 1F, G), which might suggest failures in PAR-6 trafficking or recycling.

To quantify the PAR-6 enrichment on the cortex, the cortical area was divided to anterior and posterior halves bordered by the pseudocleavage furrow. The average fluorescence intensities of each half were measured. The ratio of anterior intensity to posterior intensity (A/P) was plotted (Fig. 1I). The A/P ratio in *rack-1(RNAi)* embryos was comparable to that of control embryos during polarity establishment phase and furrow initiation but was significantly lower during maintenance phase.

**Figure 2:**
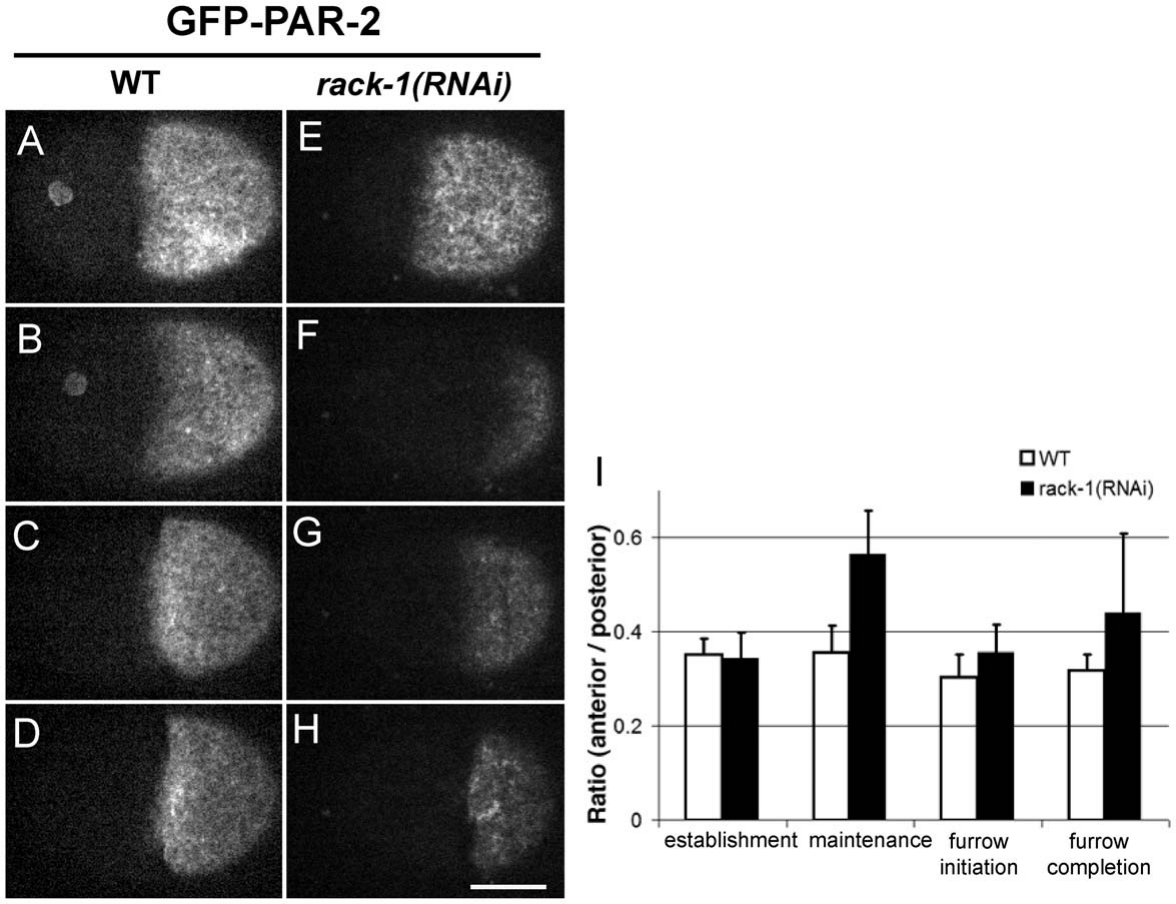
RACK-1 is required for localization of cortical PAR-2. Selected cortical projections from confocal time-lapse videos show GFP-PAR-2 localization in control embryos (A–D; Video S3) and embryos from *rack-1(RNAi)* worms (E–H; Video S4). For each video, four images are selected to represent establishment phase (pseudocleavage, A and E), maintenance phase (about seven minutes after retraction of the pseudocleavage furrow, B and F), cleavage furrow initiation (C and G), and furrow completion (D and H). In all images, anterior is to the left and posterior is to the right. (I) Quantification of the ratios between average GFP-PAR-2 intensity in the anterior and posterior halves. Four time points are measured corresponding to the four rows in A–H. White bars represent data from control embryos and black bars represent data from *rack-1(RNAi)* embryos. (Scale bar: 10 µm; error bars: SEM).

We then tested whether RACK-1 depletion affects the localization of the posterior PAR protein PAR-2. In untreated GFP-PAR-2 embryos, PAR-2 occupied the posterior half of the cortex to form a distinct domain by the end of establishment phase. This domain was stable throughout the cell cycle (Fig. 2A–D; Video S3). In *rack-1(RNAi)* embryos, the establishment of the posterior PAR-2 domain was not affected (Fig. 2E). During maintenance phase, however, the GFP-PAR-2 domain shrank to about 1/4–1/3 of the cell length in 5 out of 10 embryos (Fig. 2F; Video S4). In the rest of embryos, the PAR-2 domains were indistinguishable from those in control embryos. Prior to furrow initiation, the PAR-2 domain occupied a cortical area of similar sizes to those observed in control cells (Fig. 2G). We quantified the ratio of anterior intensity to posterior intensity for GFP-PAR-2 and found a significant difference between control and *rack-1(RNAi)* embryos during maintenance phase, consistent with that in the PAR-6-GFP experiments (Fig. 2I). Together these results suggest that RACK-1 is required for the localization of PAR proteins during maintenance phase, but not during establishment phase.

### RACK-1 is required for the asymmetric localization of DYN-1

**Figure 3:**
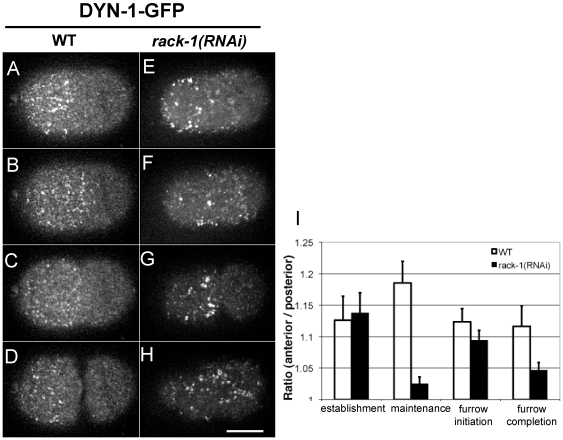
RACK-1 is required for localization of cortical DYN-1-GFP. Selected cortical projections from confocal time-lapse videos show DYN-1-GFP localization in control untreated embryos (A–D; Video S5) and embryos from *rack-1(RNAi)* worms (E–H; Video S6). For each video, four images are selected to represent establishment phase (pseudocleavage, A and E), maintenance phase (about seven minutes after retraction of the pseudocleavage furrow, B and F), cleavage furrow initiation (C and G), and furrow completion (D and H). In all images, anterior is to the left and posterior is to the right. (I) Quantification of the ratios between average DYN-1-GFP intensity in the anterior and posterior halves. Four time points are measured corresponding to the four rows in A–H. White bars represent data from control embryos and black bars represent data from *rack-1(RNAi)* embryos. (Scale bar: 10 µm; error bars: SEM).

Previous work in our lab defined a role for Dynamin/DYN-1, a large GTPase, in the maintenance of cell polarity and showed DYN-1 enrichment in the anterior cortex in a PAR-6-dependent manner [16]. Therefore we examined the consequence of RACK-1 knockdown on DYN-1-GFP localization at the cortex. Cortical Z-series projections revealed that in control embryos, DYN-1-GFP foci were enriched in the anterior half at pseudocleavage, and this anterior enrichment was stable through maintenance phase (Fig. 3A–H; also see Video S5). In *rack-1(RNAi)* embryos, the initial anterior enrichment of DYN-1-GFP foci during establishment phase was not affected (Fig. 3E; also see Video S6). However, during maintenance phase bright DYN-1-GFP puncta expanded into the posterior cortex (Fig. 3F, 7/12 embryos), and sometimes shrunk to a smaller size than that of wild type (2/12 embryos). The expansion/shrinkage was corrected with furrow initiation, and the cleavage furrow was properly placed (Fig. 3G). DYN-1 localization in the two daughter cells was normal, with the exception that in embryos with failed cytokinesis DYN-1-GFP expanded to the posterior cortex after furrow regression (Fig. 3H). We also noticed that in *rack-1(RNAi)* embryos, the DYN-1-GFP foci were clumping into larger foci than in wild type. These results suggest that RACK-1 is required for stabilizing the asymmetric localization of DYN-1 during maintenance phase.

### Astral microtubules are required for stabilizing anterior-posterior polarity

In previous studies, we have found that RACK-1 is required for RAB-11 localization [30]. RAB-11 is a small GTPase that is associated with recycling endosomes and is required for endosomal recycling [33], [34]. Interestingly, knockdown of RAB-11 displayed a similar phenotype to that of *rack-1(RNAi)*, where a smaller domain of endogenous PAR-2 during maintenance phase was observed [35]. Both *rack-1(RNAi)* and *rab-11(RNAi)* resulted in shorter astral microtubules during prometaphase, which later elongated to normal lengths during metaphase and anaphase [30], [35]. Since microtubules are required for the establishment of polarity in *C. elegans* embryos [13], [14], [22], [24], we wanted to determine whether they also play a role during the maintenance of polarity.

**Figure 4:**
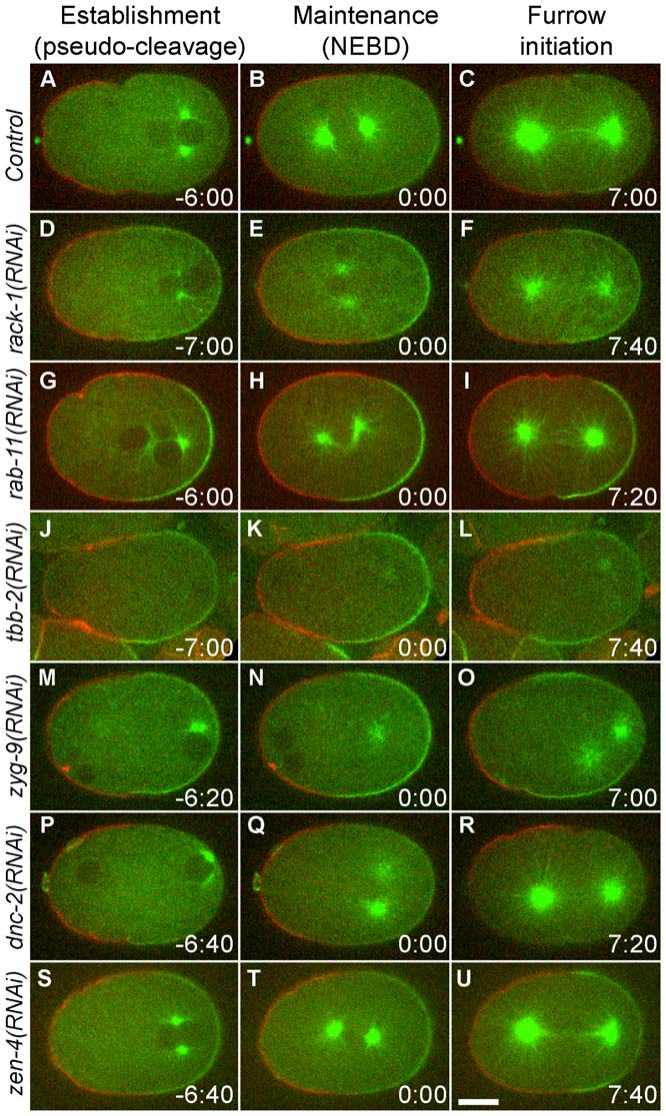
Astral microtubules are required for maintenance of polarity. Selected confocal images from time-lapse videos simultaneously show the localization of mCherry-PAR-6 (red), GFP-TBB-2 (green) and GFP-PAR-2 (green) in a control embryo (A–C; Video S7), a *rack-1(RNAi)* embryo (D–F; Video S8), a *rab-11(RNAi)* embryo (G–I; Video S9), a *tbb-2(RNAi)* embryo (J–L; Video S10), a *zyg-9(RNAi)* embryo (M–O; Video S11), a *dnc-2(RNAi)* embryo (P–R; Video S12) and a *zen-4(RNAi)* embryos (S–U; Video S13). For each video, three images are selected to represent establishment phase (last frame of pseudocleavage retraction, left column), maintenance phase (first frame after NEBD starts, middle column, time zero), and cleavage furrow initiation (right column). Time stamps are in minute:second form and in respect to NEBD. In all images, anterior is to the left and posterior is to the right. In *tbb-2(RNAi)* and *zyg-9(RNAi)* embryos, the delay of oocyte pronuclear migration results in asynchronized NEBD between paternal and maternal pronuclei. *tbb-2(RNAi)* embryos failed to form a cytokinetic furrow. (Scale bar: 10 µm).

In order to monitor PAR protein localization and microtubule dynamics simultaneously, we created a strain expressing GFP-PAR-2, mCherry-PAR-6, and GFP-TBB-2. This strain also allowed us to monitor PAR-6 and PAR-2 in the same embryo because not all of the embryos labeled with single protein were giving the same phenotype. Time-lapse images were recorded at mid-focal plane from pseudocleavage through the end of cytokinesis. In control embryos, PAR-2 and PAR-6 formed distinct cortical domains after establishment phase (hallmarked by the pseudocleavage furrow) (Fig. 4A–C; Video S7). The boundaries between the two domains were perpendicular to the AP axis and were stable throughout the cell cycle. Astral microtubules started to grow during pronuclear meeting and centration. During metaphase and anaphase the astral microtubules elongated extensively to reach the cortex.

**Figure 5:**
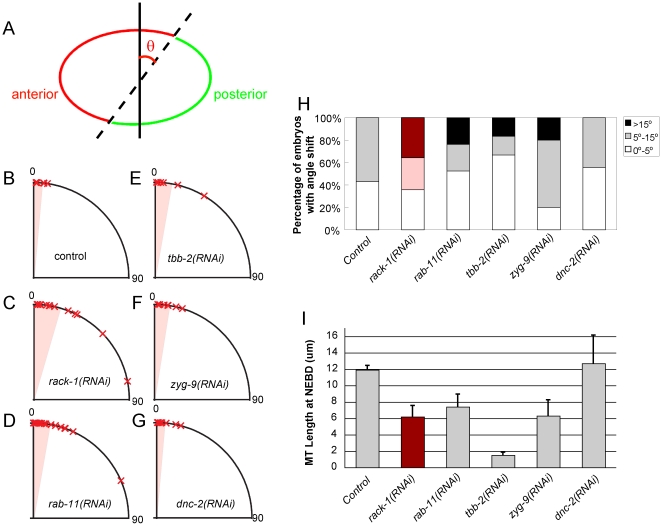
Short astral microtubule lengths correlate with polarity maintenance defects. (A) Illustration of angle shift measurement. Black line is perpendicular to the A–P axis. Dotted line represents the boundary between the PAR-6 (red) and PAR-2 (green) domains. The angle formed by these two lines is measured (θ). (B–G) Angles of PAR domain boundaries at NEBD from control (B), *rack-1(RNAi)* (C), *rab-11(RNAi)* (D), *tbb-2(RNAi)* (E), *zyg-9(RNAi)* (F), and *dnc-2(RNAi)* (G) embryos. Marks indicate individual data points, and shading indicates the mean angle of all data points. (H) The percentages of embryos with different angles were plotted. White represents embryos with minimum (less than 5 degrees) angle shift. Shading represents embryos with medium (5 to 15 degrees) angle shift. Dark represents embryos with severe (>15 degrees) angle shift. (I) The average lengths of astral microtubules were measured and plotted from untreated worms and worms with different RNAi treatments. (Error bars: SEM).

In *rack-1(RNAi)* embryos (Fig. 4D–F; Video S8), localization of PAR proteins during establishment phase was not unlike control embryos (Fig. 4D). During maintenance phase, however, the boundaries between the PAR-6- and PAR-2-occupied domains shifted and rotated to positions not perpendicular to the AP axis (Fig. 4E and 5C). Meanwhile, the astral microtubules in prometaphase were significantly shorter than those in control embryos (at NEBD, 11.9±0.6 µm in control embryos and 6.2±1.4 µm in *rack-1(RNAi)* embryos). During anaphase, the astral microtubules elongated to normal lengths (16.2±1.2 µm in control embryos and 14.1±2.2 µm in *rack-1(RNAi)* embryos). The boundaries between PAR-6 and PAR-2 rotated back to wild-type positions, and the furrow formed at the correct position (Fig. 4F). Although we occasionally observe embryos showing expansion of the PAR-6 domain and shrinkage of the PAR-2 domain in these mid-focal-plane images and in cortical images from this same strain, we did not discover a significant change in average PAR domain asymmetry between *rack-1(RNAi)* and control embryos (data not shown), which differed from phenotypes observed in PAR-6-GFP embryos and GFP-PAR-2 embryos. The strain-specific phenotypes may be due to the different expression of the PAR proteins.

We also examined the consequence of RAB-11 knockdown on polarity (Fig. 4G–I; Video S9). Similar to *rack-1(RNAi)*, *rab-11(RNAi)* led to shorter astral microtubules at NEBD (7.4±1.6 µm). Like *rack-1(RNAi)* embryos, *rab-11(RNAi)* embryos displayed rotated boundaries between PAR-6- and PAR-2-occupied domains during maintenance phase (Fig. 4H and 5D). Unlike *rack-1(RNAi)* phenotype, RAB-11 knockdown also affected establishment phase. Polarity failed to completely establish in *rab-11(RNAi)* embryos (Fig. 4G). However, in cells that formed a cleavage furrow, the furrow positions were not altered.

We then examined the requirement for normal-length astral microtubules in stabilizing polarity domains by knocking down other proteins that are known to function in microtubule length. Time-lapse videos were recorded in *tbb-2(RNAi)* embryos. Extended RNAi of TBB-2, a tubulin subunit, resulted in sterility [24]. Therefore we selected a feeding RNAi duration so that the worms could still produce embryos but were partially sterile. Under this condition, the microtubules were still nucleated at the centrosomes but failed to elongate throughout the cell cycle (1.5±0.4 µm at NEBD; Fig. 4J-L; Video S10). In these embryos, polarity was still successfully established (Fig. 4J) [11], [24] but failed to be stabilized during maintenance phase, as shown by the shift of boundaries between the PAR-6 and PAR-2 domains (Fig. 5E).

We also knocked down ZYG-9, the *C. elegans* homolog of CKAP5, which is a microtubule associated protein and is required for microtubule growth [36], [37]. In *zyg-9(RNAi)* embryos, the lengths of astral microtubules averaged 6.3±2.0 µm at NEBD. Similar to *tbb-2(RNAi)* embryos, polarity establishment was not affected, but during maintenance phase the boundaries between PAR-6 and PAR-2 were not perpendicular to the AP axis (Fig. 4M–O and 5F; also see Video S11). Together, these findings suggested that long astral microtubules are necessary for the stable localization of polarity domains during maintenance phase.

### Defects in polarity maintenance are not due to centrosome orientation or kinesin ZEN-4

We noticed that in *tbb-2(RNAi)* and *zyg-9(RNAi)* embryos, pronuclear migration and rotation were altered, which resulted in centrosomes remaining in the posterior of the embryos and failing to rotate. In addition, the migration of oocyte pronuclei was delayed. Paternal pronuclei underwent NEBD prior to pronuclear meeting and formed the spindle without maternal chromosomes. The mitotic spindle set up near the posterior, perpendicular to the AP axis. The result of this posteriorly placed spindle was the formation of two furrows, one in the anterior at the borders between the PAR-6 and PAR-2 domains, and a second furrow invaginating in the posterior between the two centrosomes (Fig. 4L,O). The presence of multiple furrows is consistent with the phenotype observed previously in worm embryos [38] and fly neuroblasts [39], presumably representing a polarity-induced and a spindle-induced furrow [39].

We tested whether these defects in centrosome orientation contributed to polarity defects. Knockdown of DNC-2, the p50/dynamitin subunit of the dynactin complex, elicited a similar phenotype as in *tbb-2(RNAi)* and *zyg-9(RNAi)* embryos (Fig. 4P-R; Video S12). In *dnc-2(RNAi)* embryos, centrosomes failed to rotate and spindles formed at the posterior [40]. However, microtubule lengths were normal (12.7±3.5 µm at NEBD; Fig. 4Q). Localization of PAR-6 and PAR-2 during maintenance phase appeared to be normal (Fig. 4Q and 5G), suggesting that the defects in centrosome orientation and posterior spindle placement are not responsible for the defect in polarity maintenance.

The kinesin ZEN-4/MKLP, which is a centralspindlin component [41], has been implicated in the polarization process of epithelial cells [42] and neuroblasts [39]. To determine if ZEN-4 plays a role in the polarity defects we observed, we examined embryos depleted of ZEN-4 by RNAi. In these embryos, the spindle midzone was absent and cytokinesis failed. *zen-4(RNAi)* did not shorten astral microtubules, and did not cause shifts of polarity domains (Fig. 4S–U; also see Video S13). These data indicate that ZEN-4 is not required for polarity maintenance.

### Defects in polarity maintenance associate with short astral microtubules

To determine the relationship between the stability of polarity domain during maintenance phase and astral microtubule length, we quantified the astral microtubule lengths at NEBD and the rotation of PAR domain boundaries for different perturbation conditions (Table 1, Fig. 5H and I). We found that the defects observed in maintenance phase were correlated with shorter astral microtubule length. When astral microtubules were shorter than control embryos (in the cases of *rack-1(RNAi)*, *rab-11(RNAi)*, *tbb-2(RNAi)*, and *zyg-9(RNAi)* embryos), the PAR domains were not stable during the maintenance phase of polarity and displayed more severely rotated boundaries. When astral microtubule lengths remained similar to control embryos (in the case of *dnc-2(RNAi)* embryos), the stability of PAR domains was not affected. These data support a role for microtubules in polarity maintenance.

**Table 1:** Short astral microtubule lengths associate with polarity maintenance defects.

	Astral MT length at NEBD (µm)	Shift of PAR domains (degree)
control	11.9±0.6	4.6±1.2
*rack-1(RNAi)*	6.2±1.4	16.7±5.9
*rab-11(RNAi)*	7.4±1.6	10.0±3.3
*tbb-2(RNAi)*	1.5±0.4	5.0±1.8
*zyg-9(RNAi)*	6.3±2.0	9.2±5.2
*dnc-2(RNAi)*	12.8±3.5	8.2±2.6

## Discussion

In the present study, we identified a role for RACK-1 in the maintenance phase of polarity to stabilize the cortical polarity domains. We revealed that the defects we observed in *rack-1(RNAi)* embryos correlate with short astral microtubules. By testing other conditions that affect the length of microtubules, we found that short astral microtubules associated with instability of PAR-6 and PAR-2 domains during prometaphase, suggesting that microtubules are necessary for polarity maintenance. Since the molecules and mechanisms involved in polarity are highly conserved among metazoans [7], [43], [44], microtubules are likely to be involved in maintaining cell polarity in other organisms.

### Localization of polarity domains relies on other mechanisms

In embryos that had shorter astral microtubules and were defective in polarity maintenance, we observed shifts of cortical domains. The boundaries were no longer perpendicular to the AP axis. However, we did not observe overlapping PAR-6 and PAR-2 domains, suggesting that microtubules may not be required for the antagonism of the PAR proteins. In addition, we did not observe polarity reversal phenotypes similar to those observed in *spd-5(RNAi)* experiments [24]. Therefore, it is likely that other mechanisms restrict the boundaries to a defined region. It is also possible that the PAR proteins themselves are adequate to maintain polarity at some level.

A recent study showed that the cytokinetic furrow is capable of repositioning the PAR domain boundaries [45]. In cells with expanded or smaller PAR-2 domains, PAR-2 is directed towards the site of cell division together with myosin cortical flow, therefore properly positioning the cleavage furrow and the PAR domain boundary. The existence of this correction mechanism could explain why the PAR domains returned to wild-type positions upon cell division in *rack-1(RNAi)* embryos.

### Microtubules are important for polarity maintenance

The myosin cortical flow mentioned above is dependent on G-alpha-mediated microtubule-cortex interactions [45]. In *C. elegans* embryos, the astral microtubules elongate to reach the cortex during prometaphase. *rack-1(RNAi)* and *rab-11(RNAi)* reduce the prometaphase astral microtubule length and result in unstable PAR domains. Later during anaphase the microtubules continue to grow to normal length and the defect in polarity corrects itself. The plasma membrane and microtubule interaction is likely to be important for the maintenance of cortical membrane domains. One potential mechanism could be that microtubules function to deliver some important factor(s) there.

The distinct domains in polarized cells are a result of polarized trafficking of membranes and lipids [46]. Microtubules and associated motors have been suggested to deliver signaling molecules to the plasma membrane [47], [48]. In epithelial cells, the centrosome-derived microtubules and the plus-end kinesin KIF5B are required for the transport of the apical cargo protein NGFR/p75 [49]. Some microtubule motors are capable of interacting with lipid rafts at the apical plasma membrane [50]. Microtubules, particularly astral microtubules, are also regulating the cortical contractility through membrane-cytoskeletal interaction in multiple systems [51], [52]. Based on these studies, it seems that microtubules are ideal for transporting the regulators of cortical polarity in *C. elegans* embryos as well.

The identity of the signal that dictates the localization of polarity domains has not been determined. The fact that microtubules are involved in both the establishment phase and maintenance phase of polarity suggests that both processes could share similar mechanisms and molecules. A previous model suggested that the RhoGAP CYK-4 and the RhoGEF ECT-2 are involved in polarity by altering the actomyosin network [17]. CYK-4 has been shown to interact with the kinesin-like protein ZEN-4, which localizes to the plus ends of microtubules [41]. However, ZEN-4 is unlikely to be the signal for polarity, as *zen-4(RNAi)* failed to uncover polarity defects. Future identification of the signal will greatly expand our understanding of the molecular mechanism of polarization.

## Supporting Information

Video S1 **PAR-6-GFP localization in control embryos.** Cortical Z-series projection of 4 slices with a step size of 0.5 µm from a control embryo expressing PAR-6-GFP (corresponds to Fig. 1A–D).
(MOV)

Video S2 **PAR-6-GFP localization is not maintained in** ***rack-1(RNAi)*** **embryos.** Cortical Z-series projection of 4 slices with a step size of 0.5 µm from a *rack-1(RNAi)* embryo expressing PAR-6-GFP (corresponds to Fig. 1E–H).
(MOV)

Video S3 **GFP-PAR-2 localization in control embryos.** Cortical Z-series projection of 4 slices with a step size of 0.5 µm from a control embryo expressing GFP-PAR-2 (corresponds to Fig. 2A–D).
(MOV)

Video S4 **GFP-PAR-2 localization is not maintained in** ***rack-1(RNAi)*** **embryos.** Cortical Z-series projection of 4 slices with a step size of 0.5 µm from a rack-1(RNAi) embryo expressing GFP-PAR-2 (corresponds to Fig. 2E–H).
(MOV)

Video S5 **DYN-1-GFP localization in control embryos.** Cortical Z-series projection of 4 slices with a step size of 0.5 µm from a control embryo expressing DYN-1-GFP (corresponds to Fig. 3A–D).
(MOV)

Video S6 **DYN-1-GFP localization is not maintained in** ***rack-1(RNAi)*** **embryos.** Cortical Z-series projection of 4 slices with a step size of 0.5 µm from a rack-1(RNAi) embryo expressing DYN-1-GFP (corresponds to Fig. 3E–H).
(MOV)

Video S7 **Polarity domains are maintained in control embryos.** Single mid-focal plane time-series of a control embryo expressing GFP-PAR-2; GFP-TBB-2; mCherry-PAR-6 (corresponds to Fig. 4A–C).
(MOV)

Video S8 **Polarity domains are not stable in** ***rack-1(RNAi)*** **embryos.** Single mid-focal plane time-series of a *rack-1(RNAi)* embryo expressing GFP-PAR-2; GFP-TBB-2; mCherry-PAR-6 (corresponds to Fig. 4D–F).
(MOV)

Video S9 **Polarity domains are not stable in** ***rab-11(RNAi)*** **embryos.** Single mid-focal plane time-series of a *rab-11(RNAi)* embryo expressing GFP-PAR-2; GFP-TBB-2; mCherry-PAR-6 (corresponds to Fig. 4G–I).
(MOV)

Video S10 **Polarity domains are not stable in** ***tbb-2(RNAi)*** **embryos.** Single mid-focal plane time-series of a *tbb-2(RNAi)* embryo expressing GFP-PAR-2; GFP-TBB-2; mCherry-PAR-6 (corresponds to Fig. 4J–L).
(MOV)

Video S11 **Polarity domains are not stable in** ***zyg-9(RNAi)*** **embryos.** Single mid-focal plane time-series of a *zyg-9(RNAi)* embryo expressing GFP-PAR-2; GFP-TBB-2; mCherry-PAR-6 (corresponds to Fig. 4M–O).
(MOV)

Video S12 **Polarity domains are stable in** ***dnc-2(RNAi)*** **embryos.** Single mid-focal plane time-series of a *dnc-2(RNAi)* embryo expressing GFP-PAR-2; GFP-TBB-2; mCherry-PAR-6 (corresponds to Fig. 4P–R).
(MOV)

Video S13 **Polarity domains are stable in** ***zen-4(RNAi)*** **embryos.** Single mid-focal plane time-series of a *zen-4(RNAi)* embryo expressing GFP-PAR-2; GFP-TBB-2; mCherry-PAR-6 (corresponds to Fig. 4S–U).
(MOV)
